# Antiglycation of Insulin by Selected Six Bioactive Peanut Sprout Stilbenoids and Demonstration of Methylglyoxal‐Mediated Biphasic Insulin Glycation by *O*‐Phenylenediamine Derivatization

**DOI:** 10.1002/fsn3.72139

**Published:** 2026-08-05

**Authors:** Po‐Chang Chiu, Shu‐Mei Lin, Yu‐Jang Li, Chih‐Yu Lo, Robin Y.‐Y. Chiou

**Affiliations:** ^1^ Department of Food Science National Chiayi University Chiayi Taiwan; ^2^ Department of Applied Chemistry National Chiayi University Chiayi Taiwan

**Keywords:** 2‐(hydroxymethyl)‐1,2,3,4‐tetrahydroquinoxaline‐2,3‐diol (2HMQL), arachidin‐3, bovine insulin glycation, MG‐biphasic glycation, peanut sprout stilbenoids

## Abstract

Protecting insulin molecules from glycation by the dietary ingredients is critical to alleviate diabetes‐associated metabolic complications. Six peanut sprout stilbenoids were selected for antiglycation determination with bovine insulin mediated by methylglyoxal (MG), glucose, and fructose. Monitoring at 215 nm via HPLC analysis revealed that all test compounds exhibited inhibitory activities with structure–activity dependency, among those arachidin‐3 performing the most potent efficacy (*p* < 0.05). Notably, a biphasic dose–response was observed, indicating that MG‐mediated insulin modification increased as MG concentrations decreased from 500 to 5 mM and then declined as MG concentrations dropped to 0.05 mM. This was supported by insulin band‐intensity changes shown in Tricine SDS‐PAGE pattern. *O*‐phenylenediamine (OPD) was utilized as a trapping probe to form MG‐OPD adducts for characterization and isolation. In high‐MG concentrations, a novel intermediate: 2‐(hydroxymethyl)‐1,2,3,4‐tetrahydroquinoxaline‐2,3‐diol (2HMQL) was isolated, identified and merited to speculate its precursor compounds existing in the MG equilibrium complex with less reactive hydrates or tautomers (e.g., dihydroxyacetone and/or hydroxypyruvaldehyde) which protect insulin integrity from glycation. It is of merit to demonstrate that identification of 2HMQL provides a proposed equilibrium mechanism to elucidate the biphasic MG‐mediated insulin glycation.

## Graphical abstract

Graphical abstract demonstrates 6 peanut sprout containing stilbenoids exhibiting varied structure‐activity potency in protection of insulin integrity from glycation mediated by methylglyoxal (MG), glucose and fructose. Meanwhile, MG‐mediated biphasic insulin glycation was evidenced in Tricine SDS‐PAGE analysis and supported by *O*‐phenylenediamine (OPD) derivatization. Identification of 2‐(hydroxymethyl)‐1,2,3,4‐tetrahydroquinoxaline‐2,3‐diol (2HMQL) provides a proposed equilibrium mechanism with plausible presence of hydroxypyruvaldehyde (HPVA) to elucidate the biphasic insulin glycation.

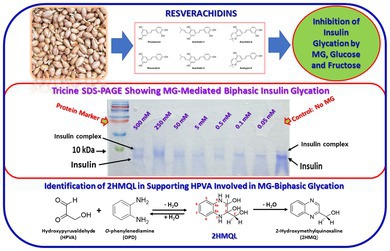

## 1 Introduction

Insulin is a dynamic and finely regulated hormone secreted by the β‐cells of pancreatic islets. It plays a crucial role in blood glucose control and participates in a wide spectrum of metabolic signaling and regulation. The insulin polypeptide in plasma or proinsulin polypeptide in pancreatic β‐cells is a target for glycation. Glycated insulin impairs metabolic regulation and drives metabolic dysfunction, leading to glucose intolerance and insulin resistance, which can underlie type 2 diabetes mellitus (T2DM) and other complications (Hunter et al. 2003; Lindsay et al. 2003; Ma et al. 2019; Walke et al. 2021; Lai et al. 2022). In patients with T2DM, ~10% of the total insulin exists in glycated form (Hunter et al. 2003; Lindsay et al. 2003). Many proteins, including insulin, exhibit a high tendency to undergo glycation during hyperglycemic conditions in patients with T2DM as well as during aging in healthy individuals (Bucala 2014). In addition to chemically modifying insulin molecules, glycation crosslinks insulin into oligomers and induces the aggregation of human islet amyloid polypeptide, a triggering factor for the onset of T2DM (Ma et al. 2019). Glycated insulin has been demonstrated to induce insulin resistance through the advanced glycation end products–receptor for advanced glycation end products signaling axis (Walke et al. 2021). Because glycation impairs insulin functionality and contributes to health deterioration, enhanced regulation of insulin glycation by searching for novel food‐originated ingredients with antiglycative activities is crucial for health management.

**FIGURE 1:**
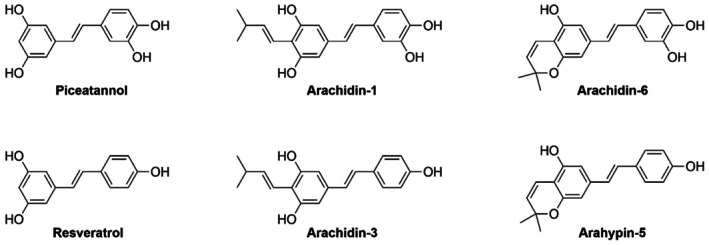
Chemical structures of three pairs of the selected six peanut sprout stilbenoids; pair 1: Piceatannol and resveratrol as simple stilbenoids; pair 2: Arachidin‐1 and arachidin‐3 as isopentadienyl stilbenoids; and pair 3: Arachidin‐6 and arahypin‐5 as *gem*‐methyl cyclic stilbenoids.

Most peanut stilbenoids belong to the secondary metabolites, originally known as phytoalexins, and could be induced by bio‐ and abiotic elicitations. Successful germination of peanut seeds is an essential step in transformation from a dormant seed to create and sustain a new life for the next generation. During germination, a broad spectrum of phytoalexins is necessarily biosynthesized. As conducted from the point of view of sprout vegetables, the germinated peanut kernels bear higher resveratrol content and antioxidant activities than the un‐germinated ones, and the sprouts could be categorized as a functional vegetable with toxicological acceptance (Wang et al. 2005; Lin et al. 2008). Anti‐obesity effects of peanut sprout extracts through regulation of PPAR gamma and adiponectin in adipose tissue of the high fat‐fed rats have been reported (Kang et al. 2014). Biosynthesis of the antioxidant and anti‐inflammatory stilbenoids could be largely enhanced by subjection of the peanut kernels to germination combined with mechanical wounding‐stress elicitation, and, after dehydration, defatting and pulverization, the prepared bio‐elicited peanut sprout powder (BPSP) with allergenicity reduction and toxicological acceptance has been achieved (Chang et al. 2006; Weng et al. 2022). The peanut sprout stilbenoids, such as arachidin‐1 and arachidin‐3, exhibit potent anti‐inflammatory, anti‐cancer, immunoregulatory, phytoestrogenic, and anti‐microbial activities along with antioxidant activities (Chang et al. 2006; Djoko et al. 2007; Huang et al. 2010; Weng et al. 2016; Nichanan et al. 2021; Ortiz and Martirosyan 2024; Reed et al. 2025). As observed in a previous study in the use of bovine serum albumin (BSA) glycation by fructose as a model, potential antioxidant and antiglycative activities of seven BPSP‐containing stilbenoids have been demonstrated (Chiou et al. 2017). This study extended these findings, and bovine insulin instead of BSA was subjected to assessment of antiglycation mediated by methylglyoxal (MG), glucose, and fructose. The selected compounds (Figure 1) were categorized into three distinct pairs based on structural traits, including pair 1: piceatannol and resveratrol as simple stilbenoids; pair 2: arachidin‐1 and arachidin‐3 as isopentadienyl derivatives of the pair 1 compounds; and pair 3: arachidin‐6 and arahypin‐5 as *gem*‐methyl cyclic derivatives of the pair 2 compounds. The antiglycative activities of the selected compounds against insulin glycation mediated by MG, glucose, and fructose were systematically investigated to compare their respective inhibitory potencies.

In addition to glucose and fructose, which are common glycation initiators, MG plays a critical role in mediating insulin glycation and subsequent complications (Schalkwijk and Stehouwer 2020; Lai et al. 2022; Berends et al. 2023; Schalkwijk et al. 2023; Vaskova et al. 2025). MG, a highly reactive dicarbonyl compound, is generated as a byproduct of endogenous metabolism of glucose, lipids, threonine, and ketone bodies (Vaskova et al. 2025). The degradation of triose phosphates during glycolysis is estimated to account for 90% of the total MG formed endogenously (Phillips and Thornalley 1993; Vaskova et al. 2025). MG is also present in food and living organisms, formed via Maillard reactions as one of the flavoring compounds in foodstuffs and beverages (Nemet et al. 2006; Wang and Ho 2012). Conversely, MG in manuka honey is appreciated as a potent anti‐bacterial agent, and its presence is regarded as a health‐enhancing quality measure (Johnston et al. 2018). MG also plays a dual, hormetic role in the context of tumor growth, switching its activity from pro‐tumor to anti‐tumor and may be considered a new biomarker for tumor cell proliferation (Nokin et al. 2017; Leone et al. 2021; Belpomme et al. 2024). Since endogenous MG is dynamically active to form hydrates and dimers which are unstable and readily detoxified into lactate by the glyoxalase enzyme system, accurately quantifying its plasma concentrations remains a challenge (Nemet et al. 2004; Krizner et al. 2009). The ambiguous bioactivity of MG (Kalapos 2021; Vaskova et al. 2025) combined with its ubiquitous role in metabolism deserves further investigation to better understand this compound and apprise health management strategies.

Herein, focused on inhibition of insulin glycation by the peanut‐originated ingredients, the six selected peanut sprout stilbenoids were subjected to in vitro examination of bovine insulin glycation mediated by three common initiators: MG, glucose, and fructose. As noticed during assessment of MG‐mediated glycation, a biphasic dose–response was observed. While MG is a well‐established glycation agent, its detailed reaction kinetics at varying concentrations remain meagerly investigated. Complex dimeric compounds could be formed by nucleophilic attack at the aldehyde rather than the ketone, which is different from glyoxal (Krizner et al. 2009). Thus, mainly due to difficulty in direct HPLC analysis of MG and its equilibrium complex, *O*‐phenylenediamine (OPD) was used not merely as a derivatizing agent, but as a chemical probe to track the MG complex to form OPD adducts to be chromatographically visualized in HPLC analysis and available for isolation and identification of the MG‐OPD adducts. This has led to success of identification of a novel adduct, 2‐(hydroxymethyl)‐1,2,3,4‐tetrahydroquinoxaline‐2,3‐diol (2HMQL), and a known 2‐hydroxymethylquinoxaline (2HMQ). Based on the discovered chemical structures, a primary frame to feature the precursors (MG complex reacted with OPD) was attempted to elucidate the observed MG‐biphasic mediation of insulin glycation.

## 2 Material and Methods

### 2.1 Preparation of Peanut Sprout Stilbenoids for Antiglycative Activity Determination

Bio‐elicited peanut sprout powder (BPSP) was prepared following the procedures reported (Chang et al. 2006; Chiou et al. 2017; Weng et al. 2022). The BPSP extract was prepared by homogenizing the powder with 10 volumes of 80% methanol (v/w), followed by ultrasonication and centrifugation at 9000 *g* for 10 min. The supernatant was filtered through a 0.45‐μm membrane filter and dried at 40°C in a Centrifugal Evaporator CES 8000 rotary vacuum evaporator (Panchum Co., Taipei, Taiwan). Regarding the BPSP‐derived stilbenoids, authentic resveratrol and piceatannol are universally accessible commercial standards with certified high purity (> 98%) and purchased from Sigma‐Aldrich Co., whereas arachidin‐1, arachidin‐3, arahypin‐5 and arachidin‐6 (3,4,5′‐trihydroxy‐6″, 6″‐dimethylpyrano [2″, 3″: 3′, 4′] stilbene) were isolated and identified from BPSP following previously reported procedures (Weng et al. 2016; Chiou et al. 2017; Gajurel et al. 2021; Cheng et al. 2021).

### 2.2 Construction of a Standard Insulin Curve Based on HPLC Quantification

The diluent for insulin solutions was prepared by mixing equal volumes of 20% dimethyl sulfoxide (DMSO in water) and phosphate‐buffered saline (PBS) (UR‐PBS001; UniRegion Bio‐Tech, Taipei, Taiwan) and designated as 10% DMSO/PBS. Commercial insulin solution (10 mg/mL from bovine pancreas; I0516; Sigma‐Aldrich Co., St. Louis, MO) was serially diluted with the 10% DMSO/PBS to prepare solutions containing 2500, 1000, 500, 250, 100, and 50 ppm (μg/mL) insulin. From each insulin solution, a 50‐μL aliquot was deposited into an Eppendorf tube and mixed well with 50 μL of 20% DMSO (in water) and 50 μL of PBS for HPLC analysis, which was conducted on an HPLC system equipped with a Hitachi L‐2130 pump, a Primaide 1420 UV detector (Hitachi Co., Tokyo, Japan), and a Supelco^R^ C18 column (250 × 4.6 mm, 5 μm; Sigma‐Aldrich Co.). Each analysis was run with a dual‐solvent system comprising methanol containing 0.1% formic acid (Solvent A) and water containing 0.1% formic acid (Solvent B). The gradient program was run as follows: 0 min, 40% A; 10 min, 60% A; 13 min, 75% A; 15 min, 85% A; 17 min, 40% A; and 20 min, 40% A. The injection volume, flow rate, and monitoring wavelength were 20 μL, 1 mL/min, and 215 nm, respectively. The detected insulin peak areas were plotted against the corresponding insulin concentrations, and a linear regression analysis was conducted to obtain a linear equation and the coefficient of determination (*R* ^2^).

### 2.3 Glycation of Bovine Insulin With Methylglyoxal, Glucose and Fructose

A solution of 1.5 M MG was prepared from commercially sourced MG fluid (M0252, 40% in water; Sigma‐Aldrich Co.) using PBS as a diluent. Solutions of 3 M glucose and 3 M fructose (G5767 and F0127; Sigma‐Aldrich Co.) were prepared in PBS. A solution of 3 mM aminoguanidine (AG; 396494; Sigma‐Aldrich Co.) dissolved in PBS was used as a known glycation inhibitor. A solution of 2.4 mg/mL bovine insulin was prepared from the source solution using PBS as a diluent. For each glycation assessment, 50 μL of insulin solution in a 1.5‐mL Eppendorf tube was mixed with 50 μL of 20% DMSO (in water) and 50 μL of AG, MG, glucose, or fructose. The reaction mixture was incubated in a thermal module at 50°C (Wang et al. 2011) to facilitate glycation. The working concentrations of each reaction component were as follows: insulin, 0.8 mg/mL; AG, 1 mM; MG, 500 mM; glucose and fructose, 1000 mM.

For HPLC analysis, 50 μL of 2.4 mg/mL bovine insulin solution was mixed with 50 μL of PBS and 50 μL of 20% DMSO (in water) and incubated at 50°C for 0, 2, 6, and 15 h. The HPLC chromatograms (Figure S1) showed that the insulin molecules remained thermally stable after 15 h of incubation at 50°C. The reactions of insulin with MG, glucose, and fructose for various intervals were also subjected to HPLC analysis. The HPLC chromatograms showed that insulin content decreased most rapidly in reactions containing MG, followed by those containing glucose and those containing fructose (Figure S2). The insulin peak height during glycation decreased to ~50% of its initial height after a 2‐h reaction with MG, a 6‐h reaction with glucose, and a 15‐h reaction with fructose.

### 2.4 Antiglycative Activity Assessment of Peanut Sprout Stilbenoids on Bovine Insulin Mediated by MG, Glucose, and Fructose

For assessing antiglycative activity, each of the six selected stilbenoids (Figure 1) was dissolved in 20% DMSO (in water) to a concentration of 300 μM. Solutions of 1.5 M MG, 3 M glucose, and 3 M fructose in PBS were used as glycation initiators, and 3 mM AG in 20% DMSO (in water) was used as a positive control of glycation inhibitor. Each antiglycative activity assessment reaction comprised 50 μL of glycation initiator, 50 μL of antiglycative reagent, and 50 μL of insulin in a 1.5‐mL Eppendorf tube. Based on our preliminary screening assessments covering 50, 100, and 200 μM parameters, and guided by an established study characterizing the comparative bioactivities and radical‐scavenging capacities of peanut sprout stilbenoids (Chiou et al. 2017), the 100 μM benchmark was selected as an optimized, scientifically validated standard concentration to enable a meaningful molecular structure–activity comparison. Herein, it was assessed by setting up individual reactions containing 100 μM stilbenoids or 1 mM AG (as a positive control), 0.8 mg/mL bovine insulin, and a glycation initiator (500 mM MG or 1000 mM glucose/fructose). Then, the reaction tubes were incubated at 50°C for 2 h with MG, 6 h in case of glucose, and 15 h in case of fructose. As a blank of control, 50 μL of 20% DMSO (in water without any inhibitor) was used. After the reaction, the tubes were rapidly cooled in an ice bath and stored at −20°C for further HPLC analysis. Antiglycative activity is expressed as the percentage decrease in integrated peak area for each inhibitor to the decrease observed in the blank of control.

### 2.5 Insulin Glycation With MG at Various Concentrations and Tricine SDS–PAGE Analysis

Tricine SDS–PAGE analysis was performed as outlined by Jiang et al. (2016) with modifications. Briefly, after the glycation reaction was completed, a 50‐μL aliquot of the reaction was mixed with 50 μL of tracking dye and heated at 95°C for 5 min. Two gels were prepared comprising 10 mL of 15% mini‐separation gel overlaid with 4 mL of stacking gel. PageRuler Plus pre‐stained protein ladder (Thermo Fisher Scientific Inc., Waltham, MA) was run concurrently as a protein marker. Samples (10 μL per well) were loaded, and electrophoresis was conducted in Tricine buffer at an initial voltage of 60 V for 20 min followed by 120 V for the remaining duration. After electrophoresis, the gels were stained, destained, and imaged.

### 2.6 Derivatization With *O*‐Phenylenediamine (OPD) as a Tracking Probe via HPLC Analysis

Due to the chemical nature of MG rendering difficulty in direct detection with a UV detector, OPD derivatization was performed to enable its detection by HPLC. A series of MG solutions (in PBS) were prepared with concentrations of 1500, 750, 250, 150, 15, 1.5, and 0.15 mM (corresponding working concentrations of 500, 250, 50, 5, 0.5, and 0.05 mM). A 50‐μL aliquot of MG solution was mixed with 50 μL of PBS and incubated at 50°C for 2 h. Then, 50 μL of 250 mM OPD (prepared in water; working concentration, 83.3 mM) (A11946.14; Thermo Fisher Scientific Inc.) was added and incubated for 10 min, followed by HPLC analysis. Concurrently, 50 μL of OPD was mixed with 100 μL of PBS and incubated at 50°C for 2 h as a control to monitor OPD stability.

To investigate the MG self‐interaction complexes, particularly at concentrations > 5 mM, and their interactions with OPD, MG solutions were prepared at 1500, 750, 150, and 15 mM. A 50‐μL aliquot of MG solution was mixed with 50 μL of PBS and preincubated at 50°C for 0, 10, 30, and 60 min. Following pre‐incubation, 50 μL of OPD solution was added to the mixture and incubated for an additional 10 min, followed by HPLC analysis.

### 2.7 Tracking of the Concentration‐Dependent MG Biphasic Effect on Insulin Glycation by *O*‐Phenylenediamine Derivatization

The concentration‐dependent biphasic effect of MG on insulin glycation using OPD derivatization and HPLC analysis monitored at 215 nm to simultaneously detect insulin, OPD, and MG–OPD adducts was conducted. Two sets of experiments were approached with and without OPD derivatization. A 50‐μL aliquot of 1.2 mg/mL insulin solution in PBS was mixed with 50 μL of MG solution prepared at 750, 150, 15, 1.5, and 0.15 mM (working concentrations of 250, 50, 5, 0.5, and 0.05 mM). The reaction mixture was incubated at 50°C for 2 h. In the first set of experiments, 50 μL of 250 mM OPD in water was added for an additional 10 min of incubation for derivatization, followed by HPLC analysis. In the second set of experiments, 50 μL of water was added for an additional 10 min of incubation, followed by HPLC analysis.

To examine the influence of MG–OPD pre‐interaction on the biphasic effect of MG on insulin glycation, 50‐μL aliquots of MG solutions at 1500, 750, 150, 15, 1.5, and 0.15 mM in PBS (working concentrations of 500, 250, 50, 5, 0.5, and 0.05 mM) were combined with 250 mM OPD solution in water (working concentration, 83.3 M). After pre‐incubation at 50°C for 10 min, 50 μL of 1.2 mg/mL bovine insulin solution in PBS was added and incubated for an additional 2 h, followed by HPLC analysis. As a control, 50 μL of PBS was substituted for 50 μL of MG solution.

### 2.8 Isolation and Structural Identification of the MG‐Adducts Involved in the Observed Concentration‐Dependent MG Biphasic Effect

Preliminary HPLC analysis of MG incubated at 50°C for > 10 min, followed by OPD addition and a further 10 min incubation, showed one major and one minor MG–OPD adduct peak (I and II), along with the OPD peak (Figure S3). To isolate the adducts corresponding to peaks I and II and elucidate their structures, a reaction stock was prepared in 10 Eppendorf tubes, each containing 0.4 mL of 1500 mM MG in PBS, 0.4 mL of PBS, and 0.4 mL of 250 mM OPD in water. The tubes were incubated at 50°C for 10 min, cooled in an ice bath, and centrifuged at 9000 *g* for 3 min. The supernatants were pooled, filtered through a 0.45‐μm membrane filter, and fractionated by medium‐pressure liquid chromatography (MPLC).

For MPLC, the sample was loaded onto an octadecylsilane Buchi Glass Column (2.6 × 10 cm) attached to a Buchi Pump Manager C‐615 with two Pump Modules C‐601 (Buchi Labortechnik, Flawil, Switzerland). MPLC was run with methanol containing 0.1% formic acid (Solvent A) and water containing 0.1% formic acid (Solvent B). The gradient program was as follows: 0–10 min, 40%–50% A; 10–40 min, 50%–60% A; 40–60 min, 60%–90% A; and 60–80 min, 90%–95% A. The flow rate was 5 mL/min, and fractions were collected with a Bio‐Rad fraction collector (Model 2110, Bio‐Rad, Hercules, CA) set at 5 mL/tube. The sample solution and each collected fraction were analyzed by HPLC using the procedure described above. The fractions corresponding to peaks I and II were pooled, diluted with three volumes of water, and loaded onto a solid‐phase extraction (SPE) column containing a C18 cartridge (5 g; Sigma‐Aldrich/Supelco Co.). The absorbed analytes were eluted with 80% methanol and further purified via semi‐preparative HPLC on a system equipped with an L‐7100 pump and L‐7420 UV/Vis detector (Hitachi Co.) with a Hyperprep HS C18 column (10 mm diameter × 25 cm length; Thermo Electron Corp. Hypersil Ltd., Cheshire, UK). Semi‐preparative HPLC was performed with an isocratic solvent of 66% methanol (containing 0.1% formic acid). The injection volume, flow rate, and monitoring wavelength were 0.2 mL, 3 mL/min, and 215 nm, respectively.

Fractions corresponding to peak I were pooled, diluted with three volumes of water, and loaded onto an SPE column containing a C18 cartridge (500 mg) for absorption. In consideration that the compounds of peaks I and II might be volatile and evaporate under vacuum drying, the absorbed analyte was first eluted with 3 mL of D_2_O to remove residual H_2_O, then with 1.2 mL of CD_3_OD. The last ~0.7 mL of the collected eluate was directly subjected to nuclear magnetic resonance (NMR) analysis. A similar procedure was employed for peak II fractions, except that the final elution was performed with 1.2 mL of CDCl_3_ and the last ~0.7 mL of the collected eluate was subjected to both NMR and mass spectrometry (MS) analyses.

For structural elucidation of the peak I compound, the CD_3_OD eluate after SPE was analyzed using a Bruker Avance III 500 MHz NMR spectrometer (Bruker BioSpin GmbH, Rheinstetten, Germany) equipped with a 5‐mm Broad Band Fluorine–Observe probe. The ^1^H NMR spectrum was measured at 298 K with an operating frequency of 500.13 MHz. The chemical shift for each hydrogen was recorded with reference to the residual solvent signal of CD_3_OD (at *δ*H 3.31). The coupling constants (*J*) were reported in Hertz (Hz). On the basis of the ^1^H NMR spectrum, the peak I compound was tentatively identified as 2‐methylquinoxaline (2MQ). To verify its identity, pooled peak I fractions after semi‐preparative HPLC were spiked with 0.2 ppm 2MQ (W‐511609; Sigma‐Aldrich Co.) and analyzed by HPLC to assess chromatographic overlap and retention‐time matching (Figure S4).

For structural elucidation of the peak II compound, the CDCl_3_ eluate by absorption and elution from SPE‐column was subjected to ^1^H, ^13^C, and heteronuclear single quantum coherence NMR spectroscopic analyses. The measurements were performed using standard Bruker pulse sequences. The ^1^H and ^13^C NMR spectra were recorded at 298 K with operating frequencies of 500.13 and 125.75 MHz, respectively. The chemical shift for each hydrogen was recorded with reference to the residual solvent signal of CDCl_3_ (at *δ*H 7.26 and *δ*C triplet 77.27 ppm), and coupling constants (*J*) were reported in Hertz (Hz).

MS analysis was conducted using a liquid chromatography–electrospray ionization (ESI)‐MS system (Thermo Finnigan, San Jose, CA) equipped with a syringe pump and an LXQ linear ion trap mass spectrometer incorporating an ESI interface. The source was operated in positive ion mode at 3.2 kV. Nitrogen served as sheath gas (5 arbitrary units) and auxiliary gas (0.1 arbitrary units). The capillary temperature, capillary voltage, and tube‐lens offset were 200°C, 5 V, and 70 V, respectively. The scanning range of mass‐to‐charge ratios was *m/z* 50 to *m/z* 200. Data acquisition and processing were performed using Xcalibur software (version 2.06). Purified samples were dissolved in methanol and analyzed via flow injection. In positive‐ion mode, a molecular‐ion peak was detected at *m/z* 196.94, consistent with the NMR‐elucidated structure of peak II compound with 196 Da of molecular weight.

### 2.9 Determination of 2‐Hydroxymethylquinoxaline as a Subsequent Adduct

The peak II compound was tentatively identified as 2‐hydroxymethyl‐1,2,3,4‐tetrahydroquinoxaline‐2,3‐diol (2HMQL). 2HMQL was regarded as an adduct prone to be dehydrated into 2‐hydroxymethylquinoxaline (2HMQ) or decomposed into OPD and MG‐like undetectable compounds. To verify its identity, the peak II fractions pooled after semi‐preparative HPLC were incubated at 50°C overnight and spiked with purchased 2‐hydroxymethylquinoxaline (2HMQ) (Cas. 41241‐94‐8; Angene Chemical, Nanjing, China), followed by HPLC analysis to examine RT matching and peak overlapping.

### 2.10 Statistics

Data are expressed as means ± standard deviations of triplicates. Statistical analysis was conducted using SPSS software (International Business Machines Corporation, NY, USA), including one‐way analysis of variance followed by significance analysis by Duncan's multiple range method (*p* < 0.05).

## 3 Results and Discussion

### 3.1 Chemical Characteristics of the Selected Peanut Sprout Stilbenoids

The chemical structures of the selected peanut sprout stilbenoids are illustrated in Figure 1. To systematically evaluate their respective inhibitory potencies and investigate potential structure–activity relationships, the selected compounds were categorized into three distinct pairs based on their unique structural traits: pair 1 includes piceatannol and resveratrol as simple stilbenoids differing with one hydroxy group; pair 2 comprises arachidin‐1 and arachidin‐3, which serve as the isopentadienyl derivatives of the pair 1 compounds; and pair 3 involves arachidin‐6 and arahypin‐5 as the *gem*‐methyl cyclic derivatives of the pair 2 compounds. This structural co‐relationship featured in the selected candidates provides an ideal molecular platform for an intensive comparative antiglycation profile.

### 3.2 Standard Curve for Bovine Insulin Quantification Determined Using HPLC

**FIGURE 2:**
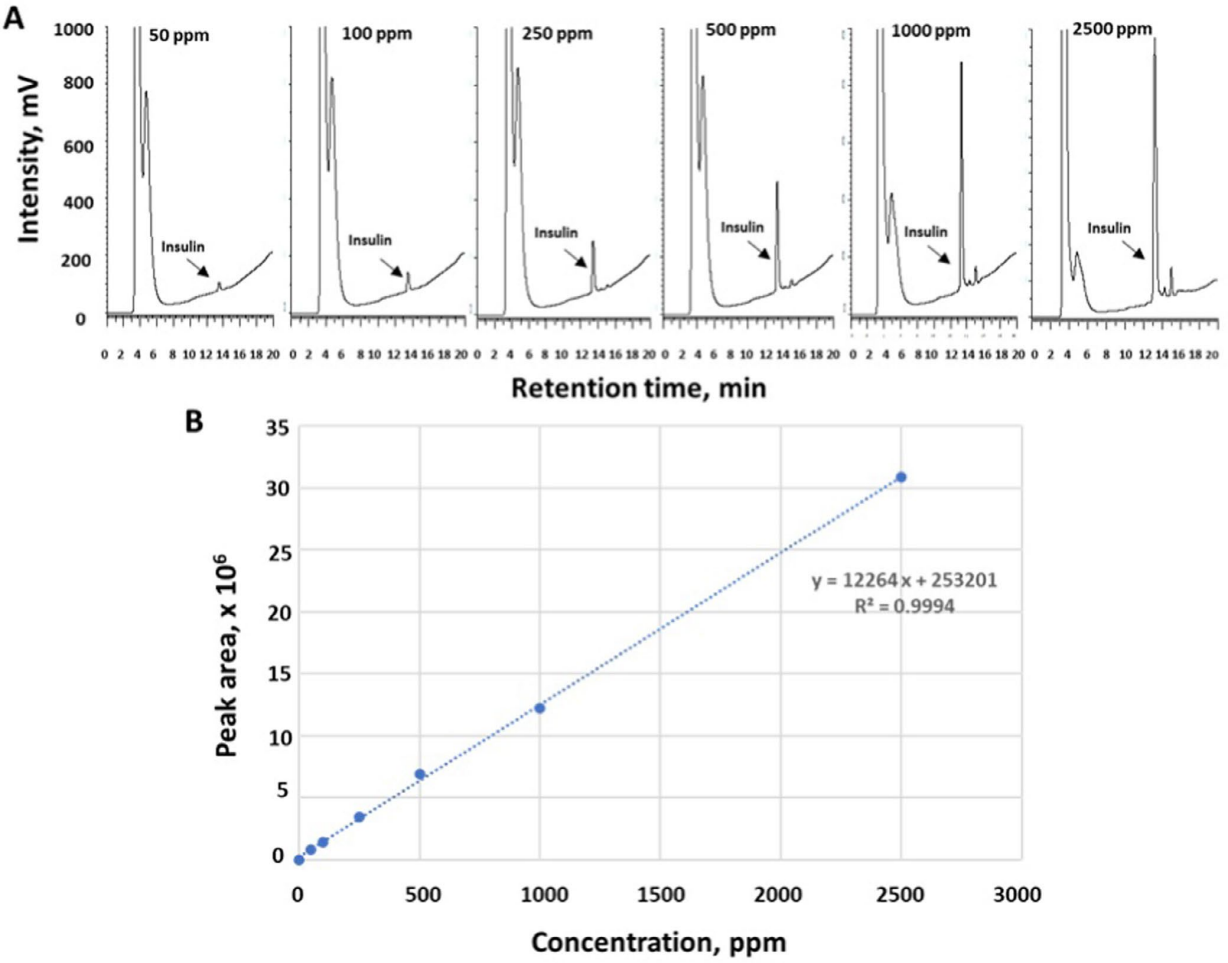
Quantification of bovine insulin by HPLC analysis. (A) HPLC chromatograms monitored at 215 nm for bovine insulin solutions ranging from 50 to 2500 ppm (μg/mL). (B) Linear regression of bovine insulin concentrations versus corresponding peak areas. Insulin peaks are indicated by arrows.

Insulin solutions prepared in 10% DMSO/PBS with concentrations ranging from 0 to 2500 μg/mL (ppm) were analyzed by HPLC. An insulin‐specific peak with RT of ~13 min was detected. Figure 2 shows the chromatograms monitored at 215 nm and the linear regression equation derived from peak areas plotted against insulin concentrations. Several protocols have been reported for insulin quantification by HPLC on C18 columns using different mobile phases (Sarmento et al. 2006; Najjar et al. 2014; Nenni 2021; Emami et al. 2022). Our laboratory protocol achieved reliable insulin detection at 215 nm within 20 min using a dual‐solvent system of methanol and water, both acidified with formic acid. The *R* ^2^ coefficient of the linear regression was 0.9994, confirming the practicality and utility of this HPLC‐based insulin quantification protocol.

### 3.3 Glycation of Bovine Insulin Using MG, Glucose and Fructose as Initiators

To facilitate glycation and save time, the glycation reaction was performed at 50°C (Wang et al. 2011), bovine insulin was separately incubated with each of the three selected glycation initiators at 50°C for varying durations. Insulin molecules with absence of glycation initiators exhibited considerable stability at 50°C for 2 and 6 h, and even after 15 h of incubation, the insulin peak area in the chromatogram decreased only slightly (Figure S1). Figure S2 and Figure 2 show the HPLC chromatograms obtained after glycation reactions at 50°C between 0.8 mg/mL bovine insulin and 500 mM MG or 1000 mM glucose/fructose for varying intervals. The insulin‐specific peak area decreased with time in all three reactions. The insulin peak height decreased by ~50% after glycation with MG for 2 h, with glucose for 6 h, and with fructose for 15 h (Figure S2), indicating that the rate of decrease was greatest with MG as the initiator, followed by glucose and then fructose. MG is known to be a highly active glycation or carbonylation precursor, promoting the formation of advanced glycation end products and increasing the risk of diabetes‐ and age‐related complications (Schalkwijk and Stehouwer 2020; Lai et al. 2022).

As shown in HPLC chromatograms (Figure S2B,C), the glucose–insulin reactions displayed pronounced peaks at RT of ~15 min, in addition to the insulin peaks while these additional peaks were faintly detected in the chromatograms of fructose–insulin reactions. These findings suggest that glucose and fructose mediate the glycation of bovine insulin through different mechanisms. Glucose and fructose are both reducing monosaccharides, and fructose is nearly 10 times more reactive than glucose in Maillard reactions, where it forms Heyns products instead of Amadori products (Gugliucci 2017). Fructose is active to glycate proteins, contributing to several T2DM complications and accelerating aging. Fructose is generally regarded as a more potent glycating agent than glucose (Bunn and Higgins 1981; McPherson et al. 1988; Suarez et al. 1989), but fructose‐mediated glycation has not been studied intensively because of the challenges involved in measuring the adducts formed (Levi and Werman 1998). Based on massive production and consumption of sucrose nowadays, the potential impact of glucose and fructose easily derived from sucrose hydrolysis is an urgent human health issue.

### 3.4 Antiglycative Activities of the Selected Peanut Sprout Stilbenoids

Antiglycative activities of the stilbenoids were calculated on the basis of the changes in integrated insulin peak areas (Table 1). As a preliminary experiment, 50 μL of 300 μM stilbenoid or 50 μL of 3 mM AG in 20% DMSO was mixed with 50 μL of 2.4 mg/mL insulin in PBS and 50 μL of PBS (without glycation initiator). The reaction mixtures were incubated at 50°C for 2 h, followed by HPLC analysis. The chromatograms showed no changes in insulin peak size (data not shown), indicating that direct interactions between insulin and each of the selected stilbenoids did not result in a consequential effect on insulin content. Since bovine insulin exhibits a highly specific, reproducible, and linear UV‐extinction profile detected by HPLC monitored at 215 nm (Figure 2), quantitative reduction of the original insulin‐specified peak area, mainly due to molecular modification driven by glycation as affected by the introduced stilbenoids, was used as a base to evaluate their antiglycative activities.

**TABLE 1:** Antiglycative activities of 1 mM aminoguanidine and 100 μM piceatannol, resveratrol, arachidin‐1, arachidin‐3, arachidin‐6, and arahypin‐5 in interaction with bovine insulin at 50°C with 500 mM methylglyoxal for 2 h, 1000 mM glucose for 6 h, or 1000 mM fructose for 15 h as glycation initiators.

Inhibitors	Glycation initiators and antiglycative activity (%)^a^, ^b^		
	Methylglyoxal	Glucose	Fructose
Aminoguanidine	10.6 ± 3.1^B^	16.8 ± 0.1^C^	41.9 ± 2.4^C^
Piceatannol	1.5 ± 0.9^D^	20.0 ± 1.6^B^	3.0 ± 1.5^D^
Resveratrol	7.6 ± 3.8^bc^	2.7 ± 1.1^E^	3.6 ± 3.1^D^
Arachidin‐1	6.3 ± 2.3^C^	20.0 ± 1.5^B^	63.0 ± 2.5^B^
Arachidin‐3	18.9 ± 0.7^A^	33.8 ± 3.0^A^	77.0 ± 4.9^A^
Arachidin‐6	3.7 ± 2.2^D^	3.0 ± 2.6^E^	46.6 ± 2.6^C^
Arahypin‐5	5.3 ± 2.3^CD^	12.3 ± 1.5^D^	2.7 ± 1.7^D^

^a^ Mean of triplicates with standard deviation (*n* = 3).

^b^ In each column, values with different superscripted capital letters indicate significant differences at *p* < 0.05.

When MG was used for glycation, MG at 500 mM is undoubtedly highly reactive, requiring only 2 h to complete one antiglycative assay. Even the antiglycative activities of the selected stilbenoids were low and distributed in a narrow range (Table 1), arachidin‐3 exhibited the highest activity (*p* < 0.05). Antiglycative activity of arachidin‐3 at 100 μM is 18.9%, which is significantly higher than that of aminoguanidine (AG) at 1 mM, a positive control of antiglycative reagent with 10.6% of activity. As further compared, resveratrol exhibited the equivalent antiglycative activity as that of AG, and both exhibited significantly higher activities than the other test compounds. Even the detailed antiglycative mechanism for each of the test stilbenoids is unclear; they have interacted with MG and/or bound with insulin molecules is inevitable. When glucose was used for glycation, arachidin‐3 exhibited the highest antiglycative activity and was even higher than that of AG at 1 mM. The antiglycative activities of resveratrol and arachidin‐6 were comparatively lower. As done with fructose used for glycation, the highest antiglycative activity was demonstrated by arachidin‐3, followed by arachidin‐1, arachidin‐6, AG, resveratrol, piceatannol, and arahypin‐5. This pattern of antiglycative activities of the test stilbenoids was in agreement with that observed in a previous study (Chiou et al. 2017), wherein fructose was incubated for glycation with bovine serum albumin (BSA).

As a general comparison among the glycation initiators, MG‐, glucose‐ and fructose‐mediated glycation proceeding through different mechanisms, it is undoubted that each test stilbenoid exhibits unique antiglycative activity according to its structure–activity relationship. As comparatively noticed, arachidin‐3 exhibited the highest activity in MG‐mediated glycation. In comparison based on chemical structures in glucose‐ and fructose‐mediated glycation, arachidin‐3 and arachidin‐1 performed higher antiglycative activity than that of resveratrol and piceatannol. This could be attributed to the presence of an extra isopentadienyl moiety at the C′‐4 position in arachidin‐3 and arachidin‐1 (Figure 1). In further comparison of arachidin‐6 and arahypin‐5 both are *gem*‐cycled stilbenoids, arachidin‐6 exhibited higher antiglycative activity in fructose‐mediated glycations than in glucose‐mediated glycation, whereas arahypin‐5 exhibited slightly higher antiglycative activity in glucose‐mediated glycation than in fructose‐mediated glycation. All the test compounds have the basic stilbene skeletal backbone, with differences either in *gem*‐cyclization or the number of attached hydroxyl or isopentadienyl groups. Therefore, the structure–activity relationships involved in insulin glycation are complex and ambiguous, warranting more intensive mechanistic investigation.

### 3.5 Concentration‐Dependent Biphasic Effect of MG on Insulin Glycation

**FIGURE 3:**
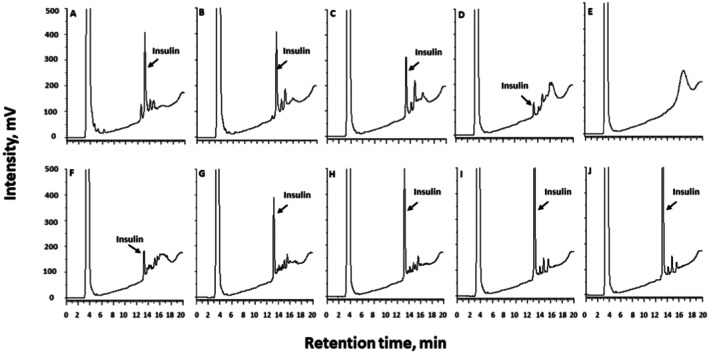
HPLC chromatographic indication of MG‐mediated glycation of bovine insulin after incubation at 50°C with various concentrations of MG for 2 h. Working concentrations: (A) 500 mM; (B) 250 mM; (C) 100 mM; (D) 50 mM; (E) 5 mM; (F) 2.5 mM; (G) 0.5 mM; (H) 0.25 mM; (I) 0.1 mM, and (J) 0.05 mM. Insulin peaks are indicated with arrows.

Insulin glycation proceeded much faster with 500 mM MG than with 1000 mM glucose or fructose was shown in Figure S2. Because MG is a highly reactive precursor that mediates advanced glycation end‐product formation, the serially diluted solutions containing 500 mM MG to 0.05 mM MG with PBS were tested for effects on insulin glycation at 50°C for 2 h, followed by HPLC analysis monitored at 215 nm (Figure 3). Unexpectedly, insulin peak size—representing non‐glycated insulin—decreased as MG concentrations decreased from 500 to 5 mM and then increased rapidly as MG concentrations decreased from 5 to 0.05 mM. This trend indicates that glycation initiation potency was highest at 5 mM MG. Contrary to the linear dose–response typically expected, a non‐monotonic “biphasic” effect where insulin glycation was most severe at 5 mM MG, rather than at 500 mM, was observed. This counter‐intuitive finding suggests that MG speciation is concentration‐dependent.

**FIGURE 4:**
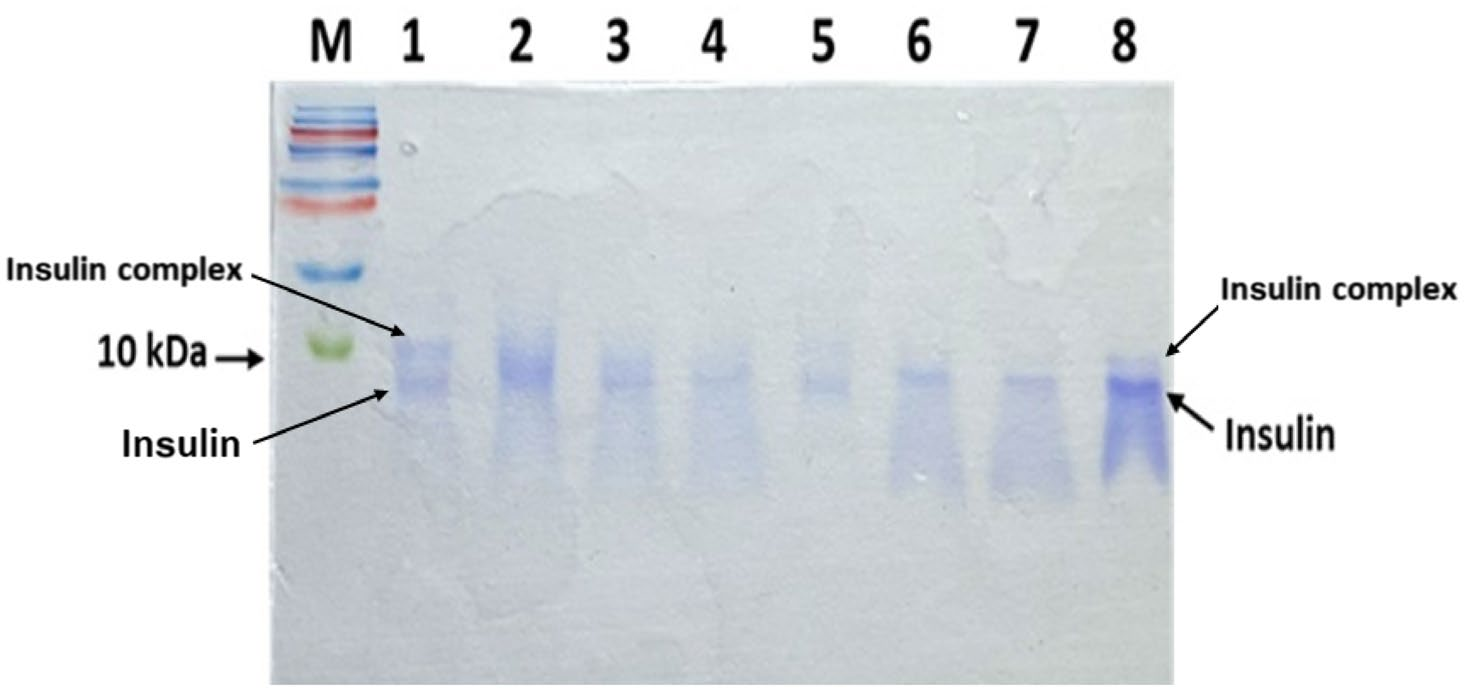
Tricine SDS–PAGE patterns of bovine insulin after incubation with various concentrations of MG at 50°C for 2 h. Protein ladder marker (M); 500 mM MG (1); 250 mM MG (2); 50 mM MG (3); 5 mM MG (4); 0.5 mM MG (5); 0.1 mM MG (6); 0.05 mM MG (7); and bovine insulin (8).

To further verify this biphasic effect of MG, the MG‐mediated insulin glycation was analyzed by Tricine SDS–PAGE (Figure 4). The stained gel displayed one major band corresponding to bovine insulin and one minor band corresponding to a higher‐molecular‐weight moiety which was designated as insulin complex in Figure 4 lane 8. In comparison, the insulin major band was detected in all test samples, and the band intensity varied with MG concentration. The band intensities decreased as MG concentration declined from 250 to 5 mM and then increased as concentration decreased further from 5 to 0.05 mM (Figure 4 lanes 2–7). For the insulin molecules glycated by MG at 500 mM (Figure 4 lane 1), a faint band being equivalent to the insulin complex of the original insulin solution shown in Figure 4 lane 8 was particularly noticed. In accordance with insulin glycation primarily monitored via HPLC at 215 nm, which detects the depletion of the native insulin peak, Tricine SDS‐PAGE was employed as an orthogonal method to visualize molecular weight shifts and cross‐linking. Both approaches were positively supporting the observed biphasic effect shown in Figures 3 and 4. The observed biphasic kinetics of MG may share characteristics with the hormetic dose–responses often observed in nutritional bioactivity (Hayes 2007, 2008), where concentration dictates the dominant chemical species and reactivity.

### 3.6 MG‐Mediated Insulin Glycation Tracked by OPD Derivatization

**FIGURE 5:**
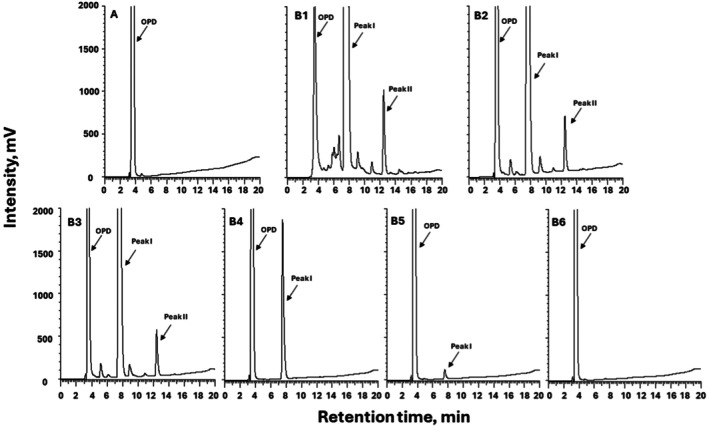
HPLC chromatograms of reactions where MG solutions of various concentrations were mixed with equal volumes of PBS and incubated at 50°C for 2 h, followed by the addition of another equal volume of 250 mM OPD in water and additional incubation for 10 min. (A) OPD incubated with two volumes of PBS for 2 h; Working concentrations: (B1) 500 mM MG; (B2) 250 mM MG; (B3) 50 mM MG; (B4) 5 mM MG; (B5) 0.5 mM MG, and (B6) 0.05 mM MG.

Due to the sophisticated and highly dynamic nature of MG‐mediated glycation, capturing the molecular shifts of uncharged or highly reactive MG clusters via conventional UV‐detection is remarkably difficult. To support our discovery of the unusual biphasic effect, we designed and executed targeted OPD‐trapping derivative analyses to capture the specific intermediates. When PBS was incubated alone at 50°C for 2 h without MG, followed by introduction of OPD for derivatization, the corresponding chromatogram displayed a single peak of OPD with RT of ~3.5 min (Figure 5A). Since DMSO strongly absorbs 215 nm and was not used in this experiment, the OPD peak was not overlapped or masked by DMSO. In addition to the OPD peak, the chromatograms of reactions containing MG at concentrations at or above 50 mM featured peaks I and II with RTs of ~7.8 and 12.8 min, respectively (Figure 5B3). The sizes of peaks I and II decreased as the MG concentration in the reaction decreased. Peak I was not detected in reactions containing 0.05 mM MG (Figure 5B6), and peak II was not detected in reactions containing 5 mM MG or below (Figure 5B4–B6). Furthermore, a lower amount of OPD remained when the reaction contained 500 mM MG as compared to 250 mM MG (Figure 5B1,B2), as evident from OPD peak size. This suggests that a greater amount of OPD was consumed to generate higher quantities of the compounds corresponding to peaks I and II in the reaction containing 500 mM MG, whereas a lower amount of OPD was consumed in the reaction containing 250 mM MG. Given that peak II was not detected in the reaction containing 5 mM MG—in which insulin glycation activity was the highest—the association between peak II and the concentration‐dependent biphasic effect of MG on insulin glycation warrants further investigation.

In followed experiments, PBS was mixed with equal volumes of MG solutions of varying concentrations (1500, 750, 150, and 15 mM). The mixture was incubated at 50°C for varying intervals (0, 10, 30, and 60 min). Subsequently, an equal volume of 250 mM OPD was added, and the mixture was incubated for an additional 10 min for derivation and followed HPLC analysis (Figure S3); in addition to peaks I and II, the chromatogram for the reaction incubated for 0 min displayed peak III with a retention time of ~14 min after OPD derivatization (Figure S3A1,B1,C1). Since peak III was detected only during the initial 30 min of incubation, it was not characterized further. Interestingly, in the chromatograms for reactions in which MG was pre‐incubated at different concentrations for 2 h prior to introduction of OPD for derivatization, the sizes of both the peaks decreased with a decrease in MG concentration (Figure 5B1–B3). Therefore, the presence of peak II being related to the concentration‐dependent biphasic effect of MG on insulin glycation was noticed; accordingly, the compounds corresponding to peaks I and II were destined for isolation and structural identification.

### 3.7 Influence of OPD Derivatization on the Biphasic Effect of MG on Insulin Glycation

**FIGURE 6:**
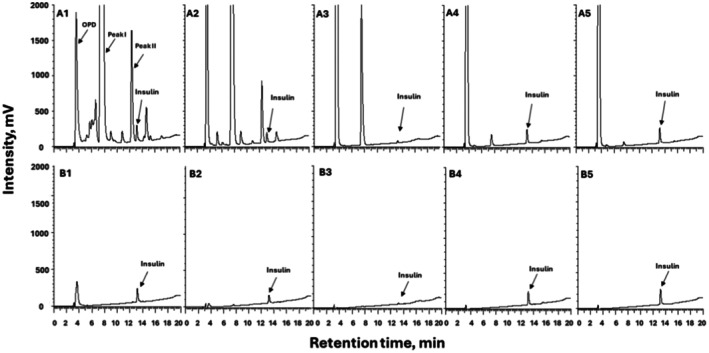
HPLC chromatographic indication of the concentration‐dependent biphasic effect of MG on bovine insulin glycation where insulin in PBS was mixed with an equal volume of MG at various concentrations and incubated at 50°C for 2 h, followed by the introduction of another equal volume of 250 mM OPD in water and an additional incubation for 10 min. (A) With OPD; (B) With water instead of OPD; Working concentrations: (1) 250 mM MG; (2) 50 mM MG; (3) 5 mM MG; (4) 0.5 mM MG; and (5) 0.05 mM MG.

Two sets of insulin solutions were prepared in PBS. Each insulin solution was mixed with an equal volume of MG solution at various concentrations and incubated at 50°C for 2 h for glycation. Then, OPD was introduced in the first set, while water was introduced in the second set in substitution of OPD, and both were incubated an additional 10 min for derivatization and HPLC analysis. As shown in Figure 6, the insulin peaks were clearly detected in both sets. Importantly, the OPD derivatization did not affect insulin detection by HPLC. Therefore, the concentration‐dependent biphasic effect of MG was evident from the decrease in insulin peak size when the MG concentration decreased from 250 to 50 mM and the subsequent increase as the MG concentration decreased further from 0.5 to 0.05 mM. The insulin peak was nearly absent in the glycation reaction with 5 mM MG, and it reappeared in the glycation reactions with 0.5 and 0.05 mM MG. It is apparent that the introduction of OPD did not influence MG‐mediated insulin glycation but served as a tracking probe to trap reactants in the MG complex to form MG‐OPD adducts to be visualized by HPLC analysis. Considering that peak II was only detected in glycation reactions containing 5 mM or above MG after OPD derivatization, the hypothesis is reasonable that an unknown compound exists in the MG complex and reacts with OPD to form the peak II compound. Thus, through the isolation and structural identification of the peak II compound, it may discover and figure out what precursor compounds exist in the MG complex and contribute to the observed biphasic effect of MG on insulin glycation.

**FIGURE 7:**
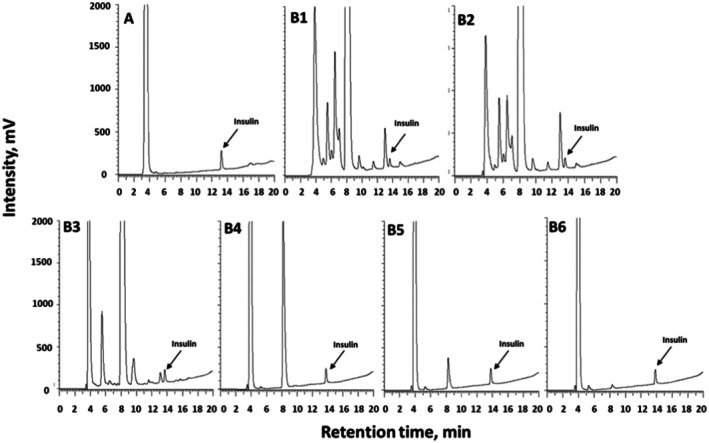
HPLC chromatograms of MG in PBS pre‐incubated with OPD at 50°C for 10 min before introducing insulin solution in PBS and an additional incubation of 2 h to induce glycation. (A) PBS (without MG) was mixed with an equal volume of OPD (250 mM in water) and pre‐incubated at 50°C for 10 min, followed by an additional incubation for 2 h with an equal volume of insulin solution. (B) MG at various concentrations was mixed with an equal volume of OPD and pre‐incubated at 50°C for 10 min, followed by an additional incubation for 2 h with another equal volume of insulin solution. Working concentrations: (B1) 500 mM MG; (B2) 250 mM MG; (B3) 50 mM MG; (B4) 5 mM MG; (B5) 0.5 mM MG, and (B6) 0.05 mM MG.

In the next experiment, MG solutions of varying concentrations in PBS were mixed with an equal volume of OPD and pre‐incubated at 50°C for 10 min for derivatization. Then, an equal volume of insulin solution prepared in PBS was introduced for an additional 2 h of incubation for glycation and HPLC analysis (Figure 7). As a control, OPD was pre‐incubated with PBS for 10 min and then insulin was introduced and incubated for an additional 2 h for glycation and HPLC analysis. The control chromatogram featured only an OPD peak with RT of ~3.5 min and an insulin peak with RT of ~13 min (Figure 7A). Insulin peaks were detected in all the reactions, indicating that the observed biphasic effect of MG was largely modified by pretreatment of MG solution with OPD. Smaller insulin peaks were observed in reactions where 250 and 500 mM MG were preincubated with 250 mM OPD, suggesting that non‐derivatized MG molecules still glycated some insulin molecules. However, the insulin peak sizes in pre‐incubation reactions containing 50, 5, 0.5 and 0.05 mM MG (Figure 7B4–B6) were close to that control (Figure 7A). Moreover, 5 mM MG—the most potent concentration for glycation—failed to glycate insulin after pre‐incubation with OPD (Figure 7B4). Thus, OPD may have reduced or impaired MG's glycation potency by modifying or destroying the precursor compounds in the MG complex, resulting in clear insulin peaks even in glycation reactions containing 5 mM MG (Figures 6B3 and 7B4). At low MG concentrations, most MG molecules are expected to be derivatized by OPD, thereby losing their glycation activity.

### 3.8 Isolation and Structural Elucidation of MG‐OPD Adducts

Based on the ^1^H NMR spectrum (Figure S5A) and referenced to the chemical shifts reported in the literature (Zubar et al. 2020), the peak I compound was tentatively identified as 2MQ (2‐methylquinoxaline). The identity of 2MQ was confirmed by spiking the collected semi‐preparative HPLC peak I fractions with commercially purchased pure 2MQ, re‐analyzing the spiked mixture by HPLC, and matching of RTs and overlapping of peaks in the resulting chromatograms (Figure S5B). Studies have reported that 2MQ is formed through direct interaction between MG and OPD (Sarmento et al. 2006; Najjar et al. 2014; Atrott et al. 2012; Nenni 2021; Emami et al. 2022).

The peak II compound was successfully isolated and purified by semi‐preparative HPLC and loaded onto an SPE C18 column. Residual H_2_O was removed with D_2_O, and the adsorbed compound was eluted with CDCl_3_ to minimize inevitable volatile loss during conventional vacuum evaporation. Finally, the compounds were subjected to MS and NMR analyses. MS analysis, conducted in the positive ESI mode, revealed a major signal at an *m/z* value of 196.94, indicating that the molecular weight of the peak II compound was 196 Da. Therefore, the peak II compound appears to be a small molecule formed simultaneously with 2MQ of the peak I compound.

**FIGURE 8:**
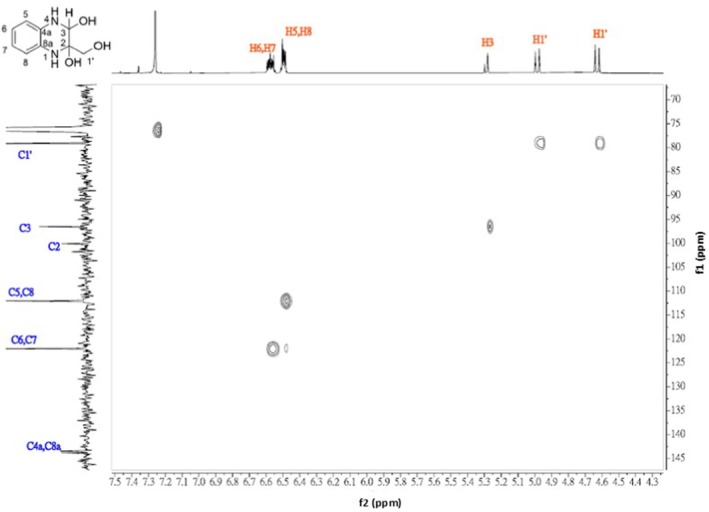
HSQC NMR spectroscopic analysis of the isolated peak II compound with a proposed structure shown in the top left corner.

As shown in Figure S5A, the ^1^H NMR spectrum of the peak II compound, along with a proposed chemical structure. The ^1^H NMR spectrum (500 MHz, chloroform‐*d*) showed chemical shifts of *δ* 6.60 (ddd, *J* = 10.3, 5.7, 3.2 Hz, 2H, H6/7), 6.52 (dd, *J* = 5.6, 3.2 Hz, 2H, H5/8), 5.01 (d, *J* = 11.6 Hz, 1H, H1′), and 4.66 (d, *J* = 11.6 Hz, 1H, H1′). The ^13^C NMR spectrum (126 MHz, chloroform‐*d*) exhibited chemical shifts of *δ* 144.7 (C), 144.3 (C), 122.9 (CH), 122.8 (CH), 113.0 (CH), 112.9 (CH), 100.9 (C), 97.3 (CH), and 80.0 (CH2) (Figure S5B). Further heteronuclear single quantum coherence analysis revealed the following relationships: (1) two H1′ were correlated with C1′; (2) H3 was correlated with C3; (3) H5 and H8 were correlated with C5 and C8; (4) H6 and H7 were correlated with C6 and C7; and (5) C2, C4a, and C8a were quaternary carbons (Figure 8). Integrating the insights obtained through NMR spectroscopy with the determined molecular weight of 196 Da, the peak II compound was identified as 2‐(hydroxymethyl)‐1,2,3,4‐tetrahydroquinoxaline‐2,3‐diol (2HMQL) (C_9_H_12_O_3_N_2_, MW = 196 Da).

**FIGURE 9:**
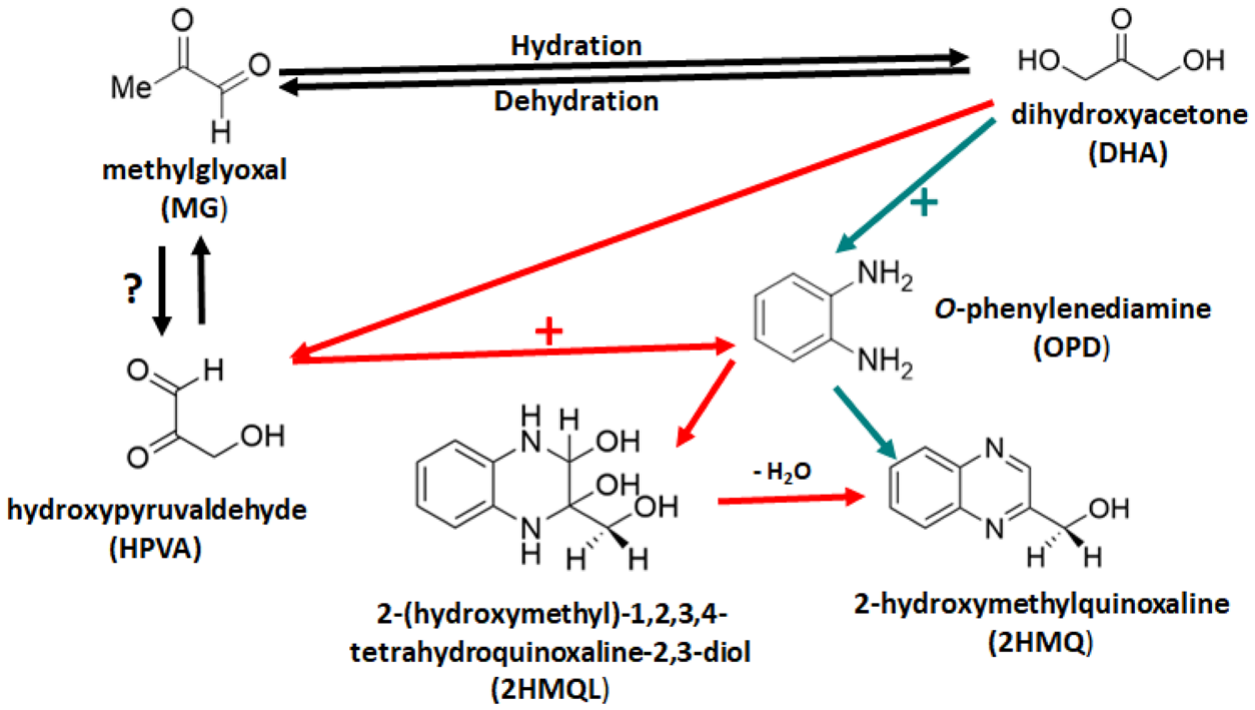
Proposed equilibrium mechanism underlying the co‐presence of DHA and HPVA with MG reacting with OPD to form 2HMQL and 2HMQ. Black arrows: Molecular changes; red arrows: 2HMQL formation; green arrows: 2HMQ formation in Manuka honey (Owens et al. 2019).

The fractions of 2HMQL collected after semi‐preparative HPLC were stored overnight at 50°C and analyzed by HPLC on the following day (Figure S6). The results indicated a decrease in the size of the 2HMQL peak, the appearance of peaks corresponding to 2HMQ (confirmed through RT matching with an authentic reference) and 2MQ (a direct MG–OPD adduct), and an increase in the size of the OPD peak. As further investigated, based on the elucidated structure, a proposed equilibrium mechanism was proposed for the generation of 2HMQL (Figure 9). 2HMQL is not a direct MG–OPD adduct; instead, it is most likely formed through the interaction between OPD and either hydroxypyruvaldehyde (HPVA; also known as 3‐hydroxy‐2‐oxopropanal or glycerosone), a dicarbonyl compound co‐present with MG, or dihydroxyacetone (DHA), a triose through glycolysis that is interchangeable with MG. HPVA can be synthesized through the oxidation of glycerol or dihydroxyacetone (Cogoli‐Greuter and Christen 1981; Thornalley and Stern 1985). Both MG and HPVA are dicarbonyl compounds that can be derived from DHA. Therefore, the generation of 2MQ and 2HMQL from MG and HPVA might follow similar modes of action, respectively. HPVA or HPVA phosphate is a natural substrate for the glyoxalase system in eukaryotic cells and is co‐present with MG in manuka honey from New Zealand (Cogoli‐Greuter and Christen 1981; Thornalley and Stern 1985; Atrott et al. 2012; Owens et al. 2019).

The direct formation of HPVA from MG follows an obscure pathway, but HPVA can be derived from DHA. Based on the chemical tracking of the OPD‐derivatized intermediates, a proposed equilibrium mechanism—rather than an absolute declaration—was formulated to describe the concentration‐dependent biphasic effect of MG on insulin glycation. While direct identification and quantification of highly active and unstable tautomeric intermediates like DHA or HPVA via standard UV‐detectors in aqueous buffers represents a major analytical challenge, the successful isolation of 2HMQL provides a valuable molecular framework to characterize the chemical species co‐existing in the MG equilibrium matrix at higher concentrations. Therefore, it is hypothesized that the co‐presence of HPVA or DHA with MG might antagonize the MG‐mediated glycation of insulin. As related, 2HMQL seems to be an OPD‐derivatized intermediate that gets readily dehydrated to form 2HMQ or decomposes to form OPD and HPVA (Figure 9). Then, the released HPVA under these conditions was transformed into MG, eventually reacting with OPD to form 2MQ.

### 3.9 Research Limitations

Despite success in comparative evaluation of the selected six peanut sprout stilbenoids on three glycation initiator platforms of MG, glucose and fructose, demonstrating structure–activity dependent potency of antiglycation of insulin, the involved details of mode of action and the affecting factors are truly complicated and deserve to be more intensively investigated. However, the observed antiglycative activities of the test peanut stilbenoids against MG‐mediated insulin glycation at 500 mM may not directly reflect the same potencies at 5 mM, which was more potent in glycation mediation (Figure 4). Tracking the true ongoing status of insulin glycation in real time is highly complex, particularly for the reactions involving MG, which undergoes rapid transformations. In this study, since native insulin can be preciously and reliably quantified via HPLC monitored at 215 nm (Figure 2), we focused on the original insulin‐specified peak changes shown in HPLC chromatograms as an objective base to evaluate the extent of glycation. To complement this, we integrated the OPD derivatization technique as a secondary probe to chemically capture and track the transient active intermediates, providing a multi‐layered validation of the ongoing reaction system. Even use of OPD derivatization enables simultaneous HPLC detection of OPD, insulin, MG‐OPD adducts and facilitates isolation and identification of 2HMQL, based to reasonably elucidate the observed MG‐biphasic glycation, developed and incorporated with advanced instruments to identify the precursor compounds existing in the MG equilibrium complex remains challenges in the future.

## 4 Conclusion

As concluded, three pairs of six selected peanut sprout stilbenoids after subjection to antiglycative characterization with bovine insulin mediated by MG, glucose and fructose and each has performed its unique structure–activity. Among those, arachidin‐3 performed the most potent efficacy of antiglycative activity (*p* < 0.05). As addressed on the MG‐mediated biphasic effect on insulin glycation, along with the successful identification of 2HMQL as a new OPD‐derivatized intermediate which provides a valuable molecular frame to figure the precursor compounds existing in the equilibrium complex of MG at or above 5 mM of concentration and proceed the observed biphasic effect on insulin glycation. The storyline was designed to bridge the gap using MG—the most active yet ambiguous small molecule in this reaction matrix. Accordingly, it is reasonable to propose an equilibrium mechanism that presence of HPVA or DHA involved with MG‐mediated glycation is most likely (Figure 9). The formation of 2HMQL requires a reversible dehydration step, indicating that at high concentrations, MG may exist reversibly in a dynamic equilibrium with the less reactive hydrates (e.g., DHA and/or tautomer of HPVA) or hydrated dimers (Krizner et al. 2009; Owens et al. 2019). Conversely, at 5 mM or lower concentrations, MG predominantly exists as reactive monomers which are capable of rapid nucleophilic attack on insulin molecules. Due to tracking the highly active and unstable tautomeric intermediates like DHA or HPVA via an UV‐detector in aqueous biological buffers represents a major analytical challenge. To ground our hypothesis, we conducted intensive and well‐designed approaches to identify the OPD‐derivatized intermediates, allowing us to propose the most plausible mechanism behind the observed biphasic effect. Both MG and HPVA are three‐carbon dicarbonyl compounds involved in carbohydrate metabolism even the free forms of MG, HPVA and DHA are hardly detected by the current analytical approaches, further investigations are required to understand whether presence of HPVA or DHA with MG leads to the formation of ambiguous complexes that hinder, interrupt, or even protect insulin molecules from MG‐mediated glycation.

## Author Contributions

**Shu‐Mei Lin:** conceptualization, writing – review and editing, supervision, data curation. **Po‐Chang Chiu:** conceptualization, investigation, writing – original draft. **Chih‐Yu Lo:** methodology, formal analysis, supervision, writing – review and editing. **Yu‐Jang Li:** methodology, formal analysis, supervision. **Robin Y.‐Y. Chiou:** conceptualization, investigation, funding acquisition, writing – review and editing, project administration.

## Funding

This research was funded by the National Council of Science and Technology, Republic of China (NSTC114‐2320‐B‐415‐001).

## Disclosure

No generative AI was used in the artworks or manuscript writing.

## Ethics Statement

The authors have nothing to report.

## Conflicts of Interest

The authors declare no conflicts of interest.

## Supporting information


**Figure S1:** HPLC chromatograms indication of thermal stability of bovine insulin after incubation at 50°C for 0, 2, 6 and 15 h in which a volume of 2.4 mg/mL of bovine insulin solution was well mixed with an equal volume of PBS and 20% DMSO and cap‐sealed for incubation at 50°C for various intervals; A1: 0 h; A2: 2 h; A3: 6 h; and A4: 15 h.
**Figure S2:** HPLC chromatograms indication of bovine insulin peak changes after interaction at 50°C with methylglyoxal (A), glucose (B) and fructose (C) for various intervals; A1: 0 h; A2: 2 h; A3: 4 h; A4: 6 h; B1: 0 h; B2: 2 h; B3: 6 h; B4: 10 h; and C1: 0 h; C2: 6 h; C3: 10 h; C4: 15 h.
**Figure S3:** HPLC chromatograms of MG solutions at various concentrations was mixed with an equal volume of PBS and subjected to various pre‐incubation at 50°C for specified intervals prior to introduction of another equal volume of OPD (250 mM in water) for additional 10 min incubation for derivatization and HPLC analysis; Working concentrations: (A) MG 500 mM; (B) MG 250 mM; (C) MG 50 mM; and (D) MG 5 mM; 1: MG was mixed with an equal volume of PBS and OPD without pre‐incubation; 2: pre‐incubation for 10 min; 3: pre‐incubation for 30 min; 4: pre‐incubation for 60 min.
**Figure S4:** (A) Chemical shift in ^1^H NMR spectroscopic analysis of the semi‐HPLC collected peak I in CD_3_OD; (B) HPLC chromatographic overlapping of the semi‐preparative HPLC collected peak I spiked with authentic 2MQ in which red: semi‐HPLC collected peak I in 67% methanol; green: 2MQ at 0.2 ppm in 80% methanol; and yellow: semi‐HPLC collected peak I spiked with 0.2 ppm of 2MQ.
**Figure S5:** Chemical shifts in ^1^H and ^13^C NMR spectroscopic analyses of the semi‐HPLC collected peak II substance dissolved in CDCl_3_; (A) ^1^H NMR; and (B) ^13^C NMR.
**Figure S6:** HPLC chromatographic changes of the semi‐preparative HPLC collected peak II fractions of 2HMQL after overnight storage at 50°C; (A) Source solution purified through semi‐preparative HPLC. (B) After storage.

## Data Availability

Data will be made available on request directed to the corresponding author.
