# Supplementary material for: Antiglycation of Insulin by Selected Six Bioactive Peanut Sprout Stilbenoids and Demonstration of Methylglyoxal‐Mediated Biphasic Insulin Glycation by O‐Phenylenediamine Derivatization
Source: Food Sci Nutr. 2026 Aug 5;14(8):e72139. doi: 10.1002/fsn3.72139 (PMC13439399; doi:10.1002/fsn3.72139)
Supplement: Supplementary file 1 [file FSN3-14-e72139-s001.docx]

**Supplementary Materials for MS (ID 1839968):**

**Antiglycation of Insulin by Selected Six Bioactive Peanut Sprout Stilbenoids and Demonstration of Methylglyoxal-mediated Biphasic Insulin Glycation by *O*-phenylenediamine Derivatization**

Po-Chang Chiu^a^, Shu-Mei Lin^a^, Yu-Jang Li^b^, Chih-Yu Lo^a^ and Robin Y.-Y. Chiou^a,1^

^a^Department of Food Science, National Chiayi University, Chiayi 60054, Taiwan ROC

^b^Department of Applied Chemistry, National Chiayi University, Chiayi 60054, Taiwan ROC

**Supplementary Figure 1**

**Supplementary Figure 1**. HPLC chromatograms indication of thermal stability of bovine insulin after incubation at 50^o^C for 0, 2, 6 and 15 h in which 50 µL of 2.4 mg/mL of bovine insulin solution was well mixed with 50 µL of PBS and 50 µL of 20% DMSO and cap-sealed for incubation at 50^o^C for various intervals; **A1**: 0 h; **A2**: 2 h; **A3**: 6 h; and **A4**: 15 h. Insulin peaks are indicated with arrows; HPLC: high-performance liquid chromatography; PBS: phosphate buffered saline; DMSO: dimethyl sulfoxide.

**Supplementary Figure 2**

**Supplementary Figure 2**. HPLC chromatograms indication of bovine insulin peak changes after interaction at 50^o^C with methylglyoxal (**A**), glucose (**B**) and fructose (**C**) for various intervals; **A1**: 0 h; **A2**: 2 h; **A3**: 4 h; **A4**: 6 h; **B1**: 0 h; **B2**: 2 h; **B3**: 6 h; **B4**: 10 h; and **C1**: 0 h; **C2**: 6 h; **C3**: 10 h; **C4**: 15 h (the insulin peaks are arrow-indicated). Insulin peaks are indicated with arrows; HPLC: high-performance liquid chromatography.

**Supplementary Figure 3**

**Supplementary Figure 3**. HPLC chromatograms of 50 µL of MG solutions at various concentrations was mixed with 50 µL PBS and subjected to various pre-incubation at 50^o^C for specified intervals prior to introduction of 50 µL OPD (250 mM in water) for additional 10 min incubation for derivatization and HPLC analysis; **A**: MG 500 mM; **B**: MG 250 mM; **C**: MG 50 mM; **D**: MG 5 mM; **1**: MG was mixed with an equal volume of PBS and OPD without pre-incubation; **2**: pre-incubation for 10 min; **3**: pre-incubation for 30 min; 4: pre-incubation for 60 min. OPD and MG-OPD peaks **I**, **II** and **III** are indicated with arrows; HPLC: high-performance liquid chromatography; MG: methylglyoxal; PBS: phosphate buffered saline; OPD: *O*-phenylenediamine.

**Supplementary Figure 4**

**Supplementary Figure 4**. **A**: Chemical shift in ^1^H NMR spectroscopic analysis of the semi-HPLC collected peak **I** in CD_3_OD**; B**: HPLC chromatographic overlapping of the semi-preparative HPLC collected peak **I** spiked with authentic 2MQ; red: semi-HPLC collected peak **I** in 67% methanol; green: 2MQ at 0.2 ppm in 80% methanol; yellow: semi-HPLC collected peak **I** spiked with 0.2 ppm of 2MQ; NMR: nuclear magnetic resonance; 2MQ: 2-methylquinoxaline.

**Supplementary Figure 5**

**A**

**B**

**Supplementary Figure 5**. Chemical shifts in ^1^H and ^13^C NMR spectroscopic analyses of the semi-HPLC collected peak **II** substance dissolved in CDCl_3_; **A**: ^1^H NMR; and **B**: ^13^C NMR.

NMR: nuclear magnetic resonance.

**Supplementary Figure 6**

**Supplementary Figure 6**. HPLC chromatographic changes of the semi-preparative HPLC collected peak **II** fractions of 2HMQL after overnight storage at 50^o^C; (**A**) Source solution purified through semi-preparative HPLC. (**B**) After storage. HPLC: high-performance liquid chromatography; OPD: *O*-phenylenediamine; 2HMQ: 2-hydroxymethylquinoxaline; 2MQ: 2-methylquinoxaline; 2HMQL: 2-(methylhydroxy)-1,2,3,4-tetrahydroquinoxaline-2,3-diol.
